# Successful low-dose azathioprine for myasthenia gravis despite hepatopathy from primary sclerosing cholangitis: a case report

**DOI:** 10.1186/1752-1947-4-356

**Published:** 2010-11-08

**Authors:** Josef Finsterer, Sonja Höflich

**Affiliations:** 1 Krankenanstalt Rudolfstiftung, Juchg. 25, 1030, Vienna, Austria

## Abstract

**Introduction:**

Although myasthenia gravis is frequently associated with other disorders, it has not been reported together with primary sclerosing cholangitis, complicating the administration of liver-toxic immunosuppressive therapy.

**Case presentation:**

A 73-year-old Caucasian woman with a history of arterial hypertension, thyroid dysfunction, glaucoma, right-sided ptosis and later generalized weakness, was diagnosed with myasthenia gravis. Additionally, primary sclerosing cholangitis was detected, initially prohibiting the administration of immunosuppressants. Despite treatment with steroids and pyridostigmine she repeatedly experienced myasthenic crises. After the fifth crisis and after antibody titers had reached levels > 100 nmol/L during two years of follow-up, it was decided to restart azathioprine. Interestingly, low-dose azathioprine (1.5 mg/kg/day) was well tolerated, had a positive clinical and immunological effect and did not worsen primary sclerosing cholangitis.

**Conclusion:**

Myasthenia gravis may occur together with primary sclerosing cholangitis in the same patient. Mild immunosuppression with azathioprine is feasible and effective in such a patient, without worsening myasthenia gravis or primary sclerosing cholangitis.

## Introduction

Myasthenia gravis (MG) may be due to a genetic defect, intoxication, or most frequently, autoimmune mechanisms [1]. Although autoimmune MG is frequently associated with other autoimmune disorders [2-13], to the best of our knowledge the association of MG with primary sclerosing cholangitis (PSC) has not been reported.

## Case presentation

**Figure 1:**
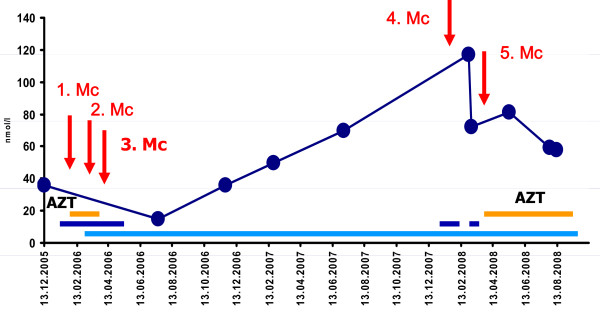
**Course of AchR-ab over almost three years, showing a positive effect of immunosuppression at onset and after the fifth myasthenic crises despite PSC**.

**Figure 2:**
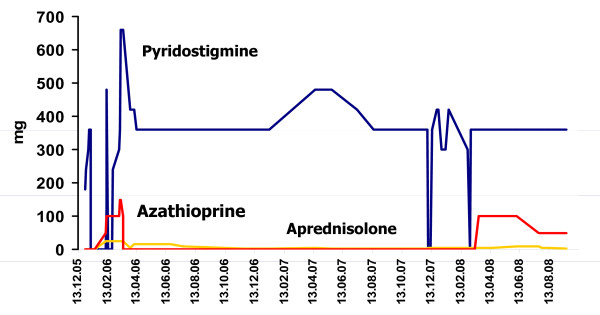
**Courses of pyridostigmine, prednisolone, and azathioprine dosages over a follow-up period of almost three years, showing that corticosteroids were given throughout this period in low dosages and that pyridostigmine dosages varied widely because of the clinical instability**.

**Figure 3:**
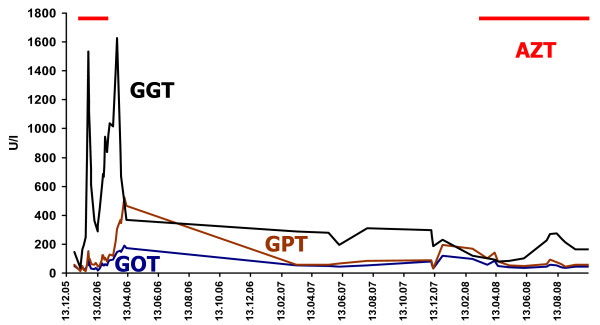
**Course of GOT *(*glutamic oxaloacetic transaminase), GPT (glutamate pyruvate transaminase) and GGT *(*gamma-glutamyl transpeptidase) over almost three years in a patient with MG and PSC**. GGT reached excessively high levels, particularly at onset of the disease, where three myasthenic crises occurred.

Our patient is a 73-year-old Caucasian woman with a history of elevated liver function parameters since age 71 and isolated right-sided ptosis one month later. Within two weeks she also developed easy fatigability, weakness when climbing stairs, ophthalmoparesis, and dysphagia for solid food, prompting hospitalization. Diagnostic work-up revealed elevated antibodies against postsynaptic acetylcholine-receptors (AchR-ab) with a titer of 35.7 nmol/L (normal < 0.4 nmol/L) (figure 1), repetitive nerve stimulation indicative of a postsynaptic transmission defect, and a positive tensilon test, so pyridostigmine and prednisolone (25 mg/day) were started (figure 2). Further diagnostic work-up revealed a mediastinal tumor and elevated liver function parameters (figure 3).

On hospital day 11 she experienced a myasthenic crisis, requiring intubation, intravenous administration of neostigmine and immunoglobulins, and plasmapheresis six times. Resection of the mediastinal tumor on hospital day 13 revealed a thymoma B3, without indication for chemotherapy. After surgery pyridostigmine was restarted but had to be replaced by neostigmine on hospital day 34 due to better efficacy. Azathioprine was initiated on hospital day 34 in a reduced dosage (50 mg/day) because of hepatopathy and increased to 100 mg/day on hospital day 44 (figure 2). On hospital day 47 she experienced a second myasthenic crisis and again required intensive care. On hospital day 57 a third myasthenic crisis manifested with dysphagia, dyspnea, and respiratory failure, again requiring intensive care. On hospital day 72, azathioprine was increased to 150 mg/day while prednisolone remained at 25 mg/day (figure 2). Upon diagnostic work-up for further increase of liver function parameters, magnetic resonance (MR)-cholangiography revealed PSC with negative anti-nuclear antibodies (ANA), smooth-muscle antibodies, anti-mitochondrial antibodies, liver-kidney antibodies, or soluble liver antigen. Ursodesoxycholic acid was given and azathioprine was discontinued (figure 2). At discharge on hospital day 108 she was under prednisolone (15 mg/day), pyridostigmine (360 mg/day), glimepiride (7 mg/day) for mild diabetes, ursodesoxycholic acid (1250 mg/day), calcium, and alendrone (70 mg once a week) for osteoporosis (figure 2). Except for right-sided ptosis, she was symptom-free.

Two months after dismissal AchR-ab reached its lowest level (figure 1) so steroids were reduced to 10 mg/day. At age 72 years, prednisolone was further reduced to 5 mg/day. Six months later she presented with right-sided ptosis, slight weakness, wasting of the thighs, exaggerated patella tendon reflexes and reduced Achilles tendon reflexes. Pyridostigmine was increased to 480 mg/day and prednisolone reduced to 2.5 mg/day. Three months later pyridostigmine was reduced to 360 mg/day without a relapse. At age 73 years she experienced a fourth myasthenic crisis during an infectious disease, requiring intubation and mechanical ventilation. After increase of prednisolone and pyridostigmine she made a full recovery. A fifth myasthenic crisis occurred five months later, which responded simply to switching from pyridostigmine to neostigmine intravenously. At that time it was decided to restart azathioprine in a dosage of 100 mg/day because of recurrent myasthenic crises and maximal elevation of AchR-ab to 117.03 nmol/L (figures 1 and 2). Because of azathioprine-induced elevation of liver function parameters (figure 3) azathioprine had to be reduced to 50 mg/day. At age 74 years corticosteroids were discontinued and azathioprine increased to 75 mg/day and later 87.5 mg/day, without further elevation of liver function parameters. Under this regimen MG did not recur and AchR-ab levels remained low until the last follow-up at age 75 years.

## Discussion

Our patient is not only interesting for the association of MG with PSC, but also for resection of a thymoma two days after onset of the first myasthenic crisis, for the transient administration of drugs, such as calcium, known to increase the risk for myasthenic crises, for the continuous rise of the AchR-ab under steroids, and for the effectiveness and tolerance of azathioprine despite its liver toxicity. Autoimmune MG is frequently associated with other autoimmune disease, including primary biliary cirrhosis (table 1), but has not been reported together with PSC as in the presented case. PSC is frequently associated with autoimmune disorders [14], such as pancreatitis, colitis ulcerosa, or Crohn's disease, of which none was found in our patient, and responds favorably to azathioprine [15]. Concerning the optimal timing of thymectomy there is general consensus that it should be carried out as soon as possible. Whether thymectomy during a myasthenic crisis may jeopardize the patient, or may prolong hospitalization and should only be carried out after pre-operative stabilization is under debate. However, there are indications that thymectomy can be safely performed even in patients with uncontrolled MG if there is proper pre-operative preparation, good anesthetic management, and optimal peri-operative respiratory care [16].

**Table 1:** Autoimmune disorders frequently associated with MG.

Disorder	Reference
Lupus erythematosus	[9]

Polymyositis	[4]

Rheumatoid arthritis	[12]

Graves' disease	[3]

Diabetes mellitus type 1	[13]

Hashimoto's thyroiditis	[3]

Scleroderma	[5]

Alopecia areata	[7]

Giant cell myocarditis	[6]

Primary biliary cirrhosis	[8]

Bronchial asthma	[10]

Addison's disease	[11]

Autoimmune pancreatitis	[2]

Despite the known intolerance and contraindications for azathioprine in PSC, it was decided to restart azathioprine because it was regarded to be effective and to have the lowest rate of side-effects among all immunosuppressants used for MG (table 2), because levels of liver function parameters were in a tolerable range, because our patient needed immunosuppressive therapy, and because the intolerability to azathioprine at age 71 years occurred during a myasthenic crisis and after surgery. Since our patient did not tolerate 100 mg/day of azathioprine, the dosage was first reduced to 50 mg/day and later to 87.5 mg/day, dosages under which AchR-ab continuously declined and no further myasthenic crises occurred during the next two years. Steroids in a low dosage were the mainstay of therapy during three years but were discontinued because they were ineffective at reducing the high levels of AchR-ab and were associated with side-effects, such as diabetes and osteoporosis. Discontinuation of steroids, which may have a favorable effect on PSC in single patients, did not worsen PSC. Why, contrary to other MG manifestations, right-sided ptosis hardly resolved, remains speculative. It is possible that she had another disorder in addition to MG, such as a multi-system metabolic disease. Overall, management of MG becomes a challenge if the patient is unstable, if AchR-ab continuously increases, and if there are contraindications for immunotherapy. However, when closely monitoring a patient for myasthenic symptoms and liver disease, it is even possible to give a liver toxic drug instead of more costly immunoglobulins or repeated plasmaphereses. The outcome may be further improved if potentially dangerous drugs in MG are avoided and close monitoring and regular re-evaluation of the medication for potential contraindications is carried out.

**Table 2:** Treatment options for primary sclerosing cholangitis.

Agent	Reference
Ursodeoxycholic acid	[17-19]

Prednisolone (initially 1 mg/kg/day)	[17-20]

Azathioprin (1-2.5 mg/kg/day)	[[17], present case, [17-21]]

Methotrexate	[22]

Tacrolimus	[18]

Endoscopic dilation of bile duct stricutres	[23]

Liver transplantation	[18,24]

## Conclusions

MG may occur together with PSC in the same patient. Immunosuppression with azathioprine in PSC and MG with progressively increasing high antibody titers is feasible, safe, and effective, even with reduced dosages, provided there is close monitoring of AchR-ab and liver function parameters.

## Competing interests

The authors declare that they have no competing interests.

## Authors' contributions

JF analyzed and interpreted the patient data regarding the blood chemical, immunological and electrophysiological investigations. SH performed some of the clinical examination and was a contributor in writing the manuscript. All authors read and approved the final manuscript.

## Consent

Written informed consent was obtained from the patient for publication of this case report and any accompanying images. A copy of the written consent is available for review by the Editor-in-Chief of this journal.
